# Neurosurgical and neuro-oncological outcomes of confirmatory brain biopsies in patients with glioblastoma: a real-life monocentric experience

**DOI:** 10.1016/j.bas.2026.105966

**Published:** 2026-02-13

**Authors:** Andrea Di Cristofori, Davide Ferlito, Francesca Graziano, Andrea Trezza, Chiara Benedetta Rui, Tommaso Calloni, Gaia Chiarello, Giovanni Stefanoni, Chiara Julita, Giovanni Palumbo, Stefania Galimberti, Giorgio Carrabba, Carlo Giussani

**Affiliations:** a Neurosurgery, Fondazione IRCCS San Gerardo dei Tintori, Via G.B. Pergolesi 33, 20900, Monza, MB, Italy; b Department of Medicine and Surgery, University of Milano-Bicocca, Ospedale San Gerardo, Milan, Italy; c Biostatistics and Clinical Epidemiology, Fondazione IRCCS San Gerardo dei Tintori, Monza, Italy; d Bicocca Bioinformatics, Biostatistics and Bioimaging Centre - B4, School of Medicine and Surgery, University of Milano-Bicocca, Italy; e Neuropathology, Fondazione IRCCS San Gerardo dei Tintori, Via G.B. Pergolesi 33, 20900, Monza, MB, Italy; f Neurology, Fondazione IRCCS San Gerardo dei Tintori, Via G.B. Pergolesi 33, 20900, Monza, MB, Italy; g Radiotherapy, Fondazione IRCCS San Gerardo dei Tintori, Via G.B. Pergolesi 33, 20900, Monza, MB, Italy; h Neuroradiology, Fondazione IRCCS San Gerardo dei Tintori, Via G.B. Pergolesi 33, 20900, Monza, MB, Italy

**Keywords:** Glioblastoma, Stereotactic biopsy, Brain MRI, Neurosurgery, Neu-ro-oncology, Radiotherapy

## Abstract

**Introduction:**

Glioblastoma (GB) is an uncurable tumor with poor prognosis despite resection *plus* adjuvant cares. When unresectable, even in case of a clear radiological imaging, guidelines require a formal histological diagnosis to confirm the diagnosis of GB.

**Research question:**

This study aims to assess the post-surgical complications and neuro-oncological outcomes of patients undergoing a confirmatory brain biopsy for diagnosing GB.

**Materials and methods:**

We considered 125 adult patients who underwent stereotactic biopsy between January 2018 and December 2023 at the Neurosurgery Department of IRCCS San Gerardo dei Tintori. Among them, 74 patients with radiological diagnosis of GB underwent a purely confirmatory biopsy. The clinical history of each patient was evaluated from the onset of symptoms through subsequent neuro-oncological treatments. We evaluated the patients' clinical conditions at the time of biopsy and upon discharge, the radiological characteristics of the tumor, the histopathological diagnosis, biopsy-related complications, access to oncological treatments along with associated complications and neurological-functional outcomes.

**Results:**

Unmethylated MGMT status, KPS≤70, tumor proximity to the internal capsule and absence of motor hemisyndrome at symptoms onset emerged as possible risk factors. Biopsied GB patients had an 11% complication rate and exhibited a dismal short-term prognosis, with a median survival of 4.7 months. Furthermore, about 40% of patients did not access subsequent treatment.

**Discussion and conclusions:**

Brain biopsy is still a minor procedure with not a negligible rate of complications. When performed as a purely confirmatory procedure, a great deal of patients does not access oncological treatments.

## Highlights

• Unresectable glioblastoma has very poor prognosis.
• Few reports about neurooncological outcome in this subgroup of patients.
• Brain biopsy is performed in order to access adjuvant treatments.
• Brain biopsy is performed also as confirmation of clear MRI findings.
• Few reports about benefits and risks of confirmatory brain biopsy.

## Graphical abstract

**Figure:**
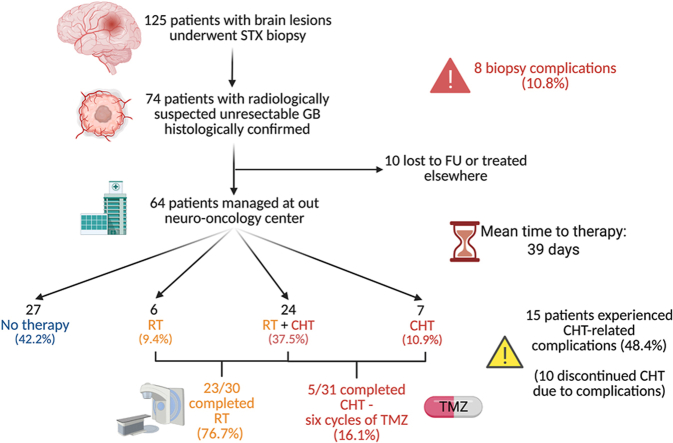

## 1 Introduction

Glioblastoma (GB) is still an uncurable malignant tumor classified as grade IV according to WHO 2021 classification (Louis et al., 2021). Its highly malignant behavior is due to the presence of glioma stem cells that proliferate in a hypoxic environment and that are highly resistant to chemo- and radiation-therapy (Yan et al., 2013). The best treatment option encompasses maximal surgical resection followed by concomitant chemoradiation therapy (with or without tumor treating fields) according to Stupp's schedule (Stupp et al., 2005; Weller et al., 2021; Olson et al., 2019; Horbinski et al., 2023). Median overall survival (OS) is 20.7 months after maximal surgical resection and adjuvant therapies (Horbinski et al., 2023; Certo et al., 2020; Hegi et al., 2005).

Surgical resection aims at a histological diagnosis and cytoreduction, while preserving neurological functions: new onset of deficits can impair the access to adjuvant therapies and it can negatively impact the Progression Free Survival (PFS), the OS and the Quality of Life (QoL). For these reasons, some GBs are considered unresectable at diagnosis due to location and/or extension of the tumor. These patients can be candidated for chemoradiation therapy only and it is known that, despite treatments, median OS is very brief (Almenawer et al., 2015; Laws et al., 2003).

In this subset of patients, provisional radiological diagnosis of GB is often confirmed with needle biopsy. Nowadays, according to guidelines by different societies, histology is still considered mandatory to formally diagnose GB and to make clinical decisions; but such recommendations are based on expert opinion only (Class IV) (Bauman et al., 2022). Such clinical conduct leads to the need for a histological diagnosis before starting any adjuvant therapy (confirmational biopsy) despite a clear radiological diagnosis.

Brain MRI has high sensitivity and specificity in distinguishing GBs from metastases and lymphoma (Suh et al., 2018; Wang et al., 2011), with increased accuracy using perfusion sequences and spectroscopy (Albert et al., 2016; Shukla et al., 2017; Corbin, 2019; Syed and Ibatullin, 2024; Zhang and Liu, 2020; Kickingereder et al., 2014). Moreover, the additional use of deep learning models is thought to increase the accuracy of radiological diagnosis in the next future (El Hachimy et al., 2024; Wisnu et al., 2024; McAvoy et al., 2021; Tariciotti et al., 2022; Swinburne et al., 2019).

A radiology-based diagnosis of central nervous system (CNS) tumors is often used in clinical practice in order to deliver adjuvant therapies like radiotherapy (RT) without a formal histological report; for example in case of meningiomas, metastases and neuromas, guidelines do not advocate for histological reports before RT or gamma-knife (Shukla et al., 2017; Xia et al., 2021).

Several surgical techniques have been described, including frameless and stereotactic biopsies (STX) (Rajshekhar, 2001; Sciortino et al., 2019); but brain biopsies are still burdened by a high complication rate (Riche et al., 2021) and, being merely diagnostic procedures, do not impact the natural history of GB. Only few authors have focused on patients’ outcomes after such procedures reporting discouraging results (Landriel Ibañez et al., 2011). Several case series concerning patients undergoing STX for intracranial lesions have been published, mostly focusing on post-operative complications. Only few cohorts of GB patients have been published (Halaj et al., 2024; Chaichana et al., 2011; Weller et al., 2023).

To our knowledge, no series exclusively concerning patients with a clear radiological diagnosis of GB undergoing confirmatory STX has been published in the post-Stupp era. Our aim was to specifically evaluate the risks and outcomes in terms of OS and access to treatment of such patients. This may be of some importance for neurosurgical and neuro-oncological counselling and management of patients requiring confirmatory biopsy only.

## 2 Materials and methods

### 2.1 Case series

#### 2.1.1 Patients’ selection

Our retrospective study encompassed consecutive patients who underwent confirmatory needle biopsy for a GB and post-operative neuro-oncological follow-up at Fondazione IRCCS San Gerardo dei Tintori (Monza, Italy) from 2018 to 2023. Follow-up period ended on the August 30, 2024. Histological diagnosis was performed according to the 2021 WHO classification of the Tumors of the Central Nervous System. Pre-2021 specimens were reviewed by a dedicated pathologist and classified accordingly.

#### 2.1.2 Inclusion and exclusion criteria

Our study included adult patients (over 18 years of age) with a clear radiological diagnosis of GB who underwent STX biopsy only for histological confirmation, needed before starting neuro-oncological treatments. None of the patients included was considered eligible for tumor resection due to site, side and extension of the tumor. Such consideration was performed by dedicated neuro-oncological surgeons during the neuro-oncology MDT. Patients in which a provisional diagnosis of GB could not be established on a pre-operative MRI were excluded, because a definitive diagnosis could only be achieved through histology. Patients that were not able to sign a consent form, without a preoperative MRI, or who underwent neuro-oncological follow-up in another institution were also excluded from this study.

#### 2.1.3 Criteria for unresectability

In our center, GB were considered unresectable following a multidisciplinary approach at the hospital tumor board. When a macroscopically significant resection could not be achieved without causing an unacceptable neurological deficit or without a real prognostic benefit, GBs were considered unresectable.

For example, morphology (e.g. mainly infiltrative behavior or anatomical locations like basal ganglia or bilateral corpus callosum without a clear mass to remove) or location (e.g. tumors in the mesencephalon, hypothalamus or tumors in the internal capsule or in the center of the motor area) of GBs are some of radiological criteria that were mainly taken into account to decide if a GB was resectable or not.

#### 2.1.4 Radiological data and criteria

All radiological and clinical records were always discussed at the neuro-oncology MDT board before any decision making. All radiological diagnoses were made by senior neuroradiologists with at least 10 years of experience in neuro-oncology.

All patients underwent a pre-operative brain MRI to assess the suspect of brain tumor, the tumor volume and the best bioptic target. The MRI protocol followed the “ideal” protocol recommendation from the standardized brain tumor imaging protocol (BTIPs) initative (Sanvito et al., 2023). An intra-axial expansile lesion was radiologically diagnosed as GB if it showed all the following “classical” features: a) non-enhancing large core, b) thick irregularly enhancing rind, c) increased cerebral blood volume (CBV) in the enhancing rind, d) T2/FLAIR hyperintensity of the adjacent whit matter (Osborn et al., 2024).

The tumor volume was calculated using the BrainLab™ segmentation software (BrainLab™, Germany).

We decided to consider proximity to the internal capsule (IC) as a speculative parameter for eloquence of tumor on motor pathways. The distance of tumor to IC was defined as the distance (in mm) between the borders of the contrast enhancing part of the tumor on the T1 with gadolinium sequences and the IC itself.

**Fig. 1:**
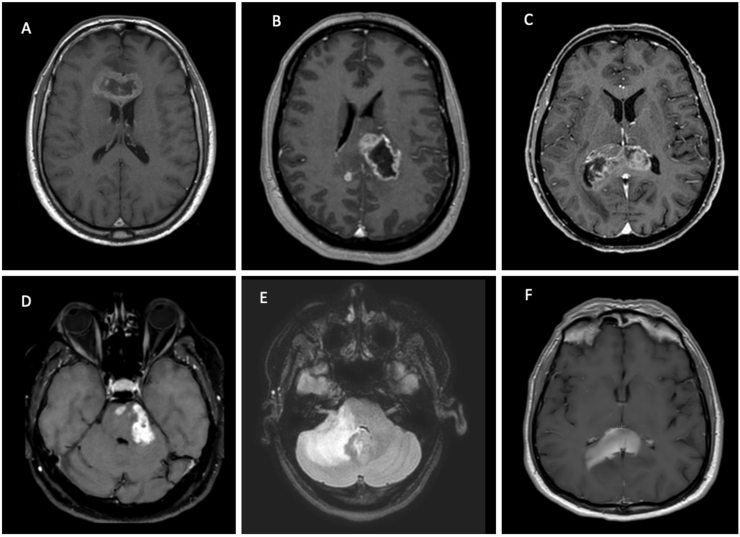
Examples of patients treated at our hospital. A,B,C) patients with IDH wild type GB that underwent a confirmatory biopsy. D) patient with cerebellar and pontine IDH wild type GB confirmed with STX biopsy. E) Patient with tumor like inflammatory disease confirmed with STX biopsy. F) Patient with primary CNS lymphoma confirmed by STX biopsy. IDH Isocitrate Dehydrogenase; GB Glioblastoma; STX Stereotactic; CNS Central Nervous System.

Fig. 1 illustrates representative cases at our institution: some patients had a presumptive radiological diagnosis of GB and therefore underwent a STX biopsy as a confirmatory procedure, whereas others required a diagnostic brain biopsy because tissue sampling was essential to achieve a definitive diagnosis.

#### 2.1.5 Data collection

Several demographic and clinical features of patients were analyzed: age, sex, symptoms/deficit at onset, gross tumor volume (GTV), distance from the IC, tumor location, number of lobes involved.

Postoperative complications were analyzed according to the previously published severity grading scale adapted to intracerebral diagnostic procedures (Landriel Ibañez et al., 2011; Mathon et al., 2020); hemorrhages causing transient symptoms (1B) were considered, but asymptomatic hemorrhages visible on the postoperative CT (1A) were not.

We then examined the access to adjuvant treatments by specifying the kind of therapy administered (RT, CHT or both). In addition, the time elapsed sinche the biopsy and the start of treatments, the number of CHT cycles, any second-line treatment (e.g. regorafenib (Wilhelm et al., 2011), fotemustine) and CHT complications were assessed. CHT complications were assessed according to Common Terminology Criteria for Adverse Events version 5.0 (CTCAE). The kind of adjuvant therapy was decided by dedicated neuro-oncologists and radiation therapists.

Patients were divided into those who started but did not complete adjuvant treatments (RT, CT or both) and those who completed them.

#### 2.1.6 Surgical procedure

At our institution, brain biopsies are performed with a frame-based system by dedicated neuro-oncological surgeons. The surgical procedure is performed under general anesthesia. After a stereotactic frame is placed, a contrast brain CT scan is performed. Imaging data are then fused with brain MRI (iPlannet Stereotaxy software, BrainLab®, Munich – Germany) to plan the target and the trajectory. Immediate post-operative CT scan for target verification and complication detection is routinely performed (Riche et al., 2021; Mathon et al., 2020).

#### 2.1.7 Neuro-oncological treatment and outcomes

Concomitant chemo-radiation therapy according to Stupp's schedule was the first line treatment (Stupp et al., 2005, 2009). Time between surgery and the first session of RT was recorded. After RT, patients were followed-up with brain MRI with gadolinium every 3 months unless new neurological events occurred (Radbruch et al., 2015; Radbruch et al., 2015; Ellingson et al., 2015). Dedicated neuro-oncologists and radiotherapists were involved in treating and following our patients.

Access to adjuvant therapies was considered successful when patients were able to complete the course of RT or to complete the first cycle of CHT in case of CHT alone. Patients who were deemed unfit for adjuvant therapies were administered best comfort care (BCC). The OS of the different patient groups in relation to access or completion of care was analyzed and compared.

### 2.2 Statistical analysis

Data are described as mean (standard deviation, SD), median (interquartile range, I-III quartiles), and absolute and relative frequencies, where appropriate Overall survival (OS) was defined as the time interval between the date of biopsy and death from any cause or last follow-up. Kaplan-Meier curves were used to estimate the median OS and the corresponding confidence Interval (95%CI). Log-rank test were used for the comparison between groups. Subsequently, Cox proportional hazard regression model was performed to assess potential risk factors on survival. In particular, the model included sex, age (above or below 65 years), MGMT promoter methylation status (methylated or unmethylated), KPS (≤70 or >70), distance from the IC (within or beyond 1 cm), GTV (above or below the median value of our cohort, i.e., 18.85 cm^3^), and initiation of any form of treatment (CHT or RT). In addition, a logistic regression model was performed to factors associated with access of oncological treatment. This model included sex, age, MGMT promoter methylation status, KPS, tumor proximity to the internal capsule, and GTV. The results were reported as hazard ratios (HRs), and as odds ratios (ORs), along with corresponding 95% CI. All tests were two-sided, with a significance level set at 0.05. Statistical analyses were performed using R software.

## 3 Results

### 3.1 Descriptive characteristics of the population

Between 2018 and 2023, 125 patients underwent stereotactic biopsy at our institution. In 51 patients radiological report was diagnostic for: diffuse glioma or other primary brain tumors in 13 cases and primary CNS lymphoma in 14 cases; in 24 cases the neuroradiologist and the neuro-oncology MDT were not able to make a clear radiological diagnosis among different neoplastic entities (e.g. glioma *vs* lymphoma) or tumoral and non-neoplastic (e.g. inflammatory disease *vs* primary CNS lymphoma). Among these cases, 9/13 (69.2%) gliomas were confirmed and 11/14 (78.6%) of primary CNS lymphomas were attested. In these 51 patients 2 (3.9%) STX biopsies resulted not diagnostic, but they were treated according to the radiological aspect (one as a brain lymphoma; one as a GB) after a multidisciplinary meeting.

These 51 patients were excluded from our study since the radiological aspect was not typical for GB. We included the remaining 74 adult patients with unresectable radiologically suspected GB who met the inclusion criteria. IDH was wild type in all patients; MGMT was methylated in 48 (64.9%) of them and unmethylated in 27 (36.5%). In 1 patient (1.4%), bioptic sample was not diagnostic and the procedure was repeated.

In Table 1 are reported the clinical and radiological features of patients included in our cohort. The mean age was 63.58 years (SD = 14.48); 35 women (47.3%) and 39 men (52.7%) were included in the study. According to the semi-automatic segmentation, the median GTV was 23.80 cm^3^ (SD = 16.81). The minimum GTV was 4.2 cm^3^ and the maximum 70.6 cm^3^ (IQR 10.93; 30.27).

**Table 1:** descriptive characteristics of population.

	Overall N = 74 n (%)
**Age** (years), mean (SD)	63.58 (14.48)
**Sex**, n (%)	
Male	39 (52.7)
Female	35 (47.3%)
**Preoperative deficit-symptoms**, n (%)	
Motor hemisyndrome	35 (46.7%)
Neurocognitive deficits	15 (20.0%)
Aphasia	4 (5.3%)
Hemianopsia	3 (4.0%)
Other	18 (24.3%)
**GTV** (cm^3^), mean (SD)	23.80 (16.21)
**IC distance** (cm), mean (SD)	0.91 (1.13)
**IC distance**, n (%)	
≥1 cm	27 (36%)
<1 cm	48 (64%)
**Tumor location**, n (%)	
Frontal	23 (30.6%)
Parietal	12 (16%)
Occipital	1 (1.3%)
Temporal	13 (17.3%)
Corpus callosum	12 (16%)
Basal ganglia	10 (13.3%)
Cerebellum	1 (1.3%)
Brainstem	3 (4%)
**Number of lobes involved**, n (%)	
1 lobe	48 (64%)
2 lobes	25 (33.3%)
≥3 lobes	2 (2.7%)
**Preoperative KPS**, mean (SD)	72.8 (11.3)
**Preoperative KPS**, n (%)	
≥70	60 (81.1%)
<70	14 (18.95%)

In 48 patients (64%), the tumor was located within 1 cm from the IC and the average distance for the whole population was 0.91 cm. In 48 (64%) patients, the tumor affected only one lobe; while in 25 (33.8%) the tumor affected two and in 2 (2.7%) more than three.

Symptoms of onset were hemiparesis in 35 cases (47.3%), cognitive impairment in 15 (20.3%), aphasia in 4 (5.4%), hemianopsia in 3 (4%) and other (e.g. seizure) in 18 (24.3%). The average KPS at the radiological diagnosis was 72.80. KPS was less than 70 in 14 (18.9%) patients.

**Fig. 2:**
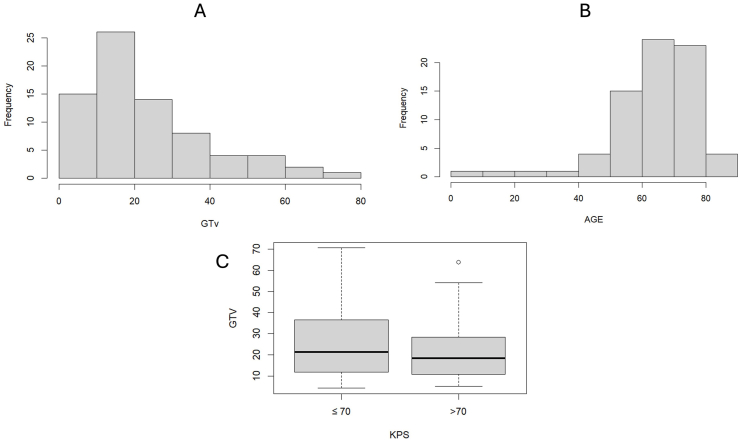
Distribution of age (A) and GTV (B) in the study population. C) Box plot illustrating the distribution of GTV stratified by KPS. GTV Gross Tumor Volume; KPS Karnofsky Performance Status.

Fig. 2 illustrates the distribution of age and GTV in the analyzed sample. It also depicts the relationship between GTV and patient performance status, stratified by a KPS score of ≤70 and > 70.

### 3.2 Post-operative complications

Eight (10.8%) patients reported at least one post-operative complication which included: 1 patient who experienced seizures and 5 patients who presented a symptomatic bleeding. Among these 5 patients, 1 of them developed a post-operative aphasia, 2 developed a new-onset hemiparesis and 2 experienced a significant deterioration in their neurological status leading to palliative care and death within 30 days of the procedure. Two (2.7%) patients experienced postoperative hemorrhages that did not result in new neurological symptoms but required an extended hospital stay for close neurological monitoring. Additionally, 1 (1.4%) patient had to undergo to an additional biopsy due to inconclusive histological diagnosis of the first one.

Eleven (14.9%) patients experienced a decline in the KPS at discharge compared to the preoperative status. We observed an average reduction of 3.5 points between preoperative and postoperative KPS scores despite no complications. See Table 2 for all biopsies complications.

**Table 2:** Biopsies complications and subsequential surgical procedure.

	Overall N = 74 n (%)
**Biopsies complications**, n (%)	
Asymptomatic bleeding	2 (2.7%)
Symptomatic bleeding	5 (6.7%)
Seizures	1 (1.3%)
**Postoperative deficit,** n (%)	
Aphasia	1 (1.3%)
Motor hemisyndrome	2 (2.7%)
Decline of consciousness	2 (2.7%)
**Subsequential surgical procedure**, n (%)	
New biopsy	1 (1.4%)
Surgical excision	4 (5.3%)
**KPS decline at discharge**, n (%)	11 (14.7%)
**Average KPS at discharge**, mean (SD)	69.3 (14.0)

### 3.3 Access to treatment and complications

Ten patients were lost to follow-up since they were treated in other centers. Twenty-seven (42.2%) patients did not undergo any kind of neuro-oncological therapy and were therefore offered best comfort cares (BCCs). These patients did not access any sort of oncological therapy due to worsening of neurological conditions while waiting for oncological therapies after a biopsy. Among those who received adjuvant treatment, 7 patients (10.9%) underwent CHT only, 6 patients (9.4%) started RT only and 24 patients (37.5%) were treated with both of them. On average, patients began treatment—whether RT or CHT—38.97 days (SD = 19.51) following stereotactic biopsy.

Twenty-three out of 30 (76.7%) patients who underwent RT completed adjuvant therapies offered. The number was significantly lower among patients who initiated CHT: only 5 out of 31 (16.1%) completed six cycles of adjuvant temozolomide, 26 (83.9%) did not due to neurological deterioration or complications arising from CHT. The mean number of temozolomide cycles administered was 2.72 (SD = 2.23). Furthermore, 8 out of 31 patients (26.7%) were offered a second-line treatment (regorafenib or fotemustine) due to radiological progression. 15 (48.4%) patients in the CHT cohort experienced at least one grade 2 CTCAE drug-related complication, such as pneumonia, pulmonary thromboembolism, hepatopathy. Details can be found in Table 3.

**Table 3:** Table summarizing the rates of access to subsequent neuro-oncological therapies, along with the associated complications and any early discontinuations.

	Overall N = 64 n (%)
**Treatment access**, n (%)	
RT	6 (9.4%)
CHT	7 (10.9%)
RT + CHT	24 (37.5%)
No therapy	27 (42.2%)
**Completation of RT**, n (%)	23/30 (76.7%)
**CHT**, n (%)	
Mean cicles of TMZ	2.72 (2.23)
Completation of CHT	5/31 (16.1%)
Early discontinuation	26/31 (83.9%)
Second line CHT	8/31 (25.8%)
**CHT complications**, n (%)	15/31 (48.45)
Steroid-induced diabetes	3 (9.7%)
Myelosuppression	10 (32.3%)
Hepatopathy	1 (3.2%)
Pneumonia	5 (16.1%)
Others	4 (12.9%)
**Mean time to initiation of therapy**, days (SD)	38.96 (19.51)

### 3.4 Prognostic factors and neuro-oncological outcome

**Fig. 3:**
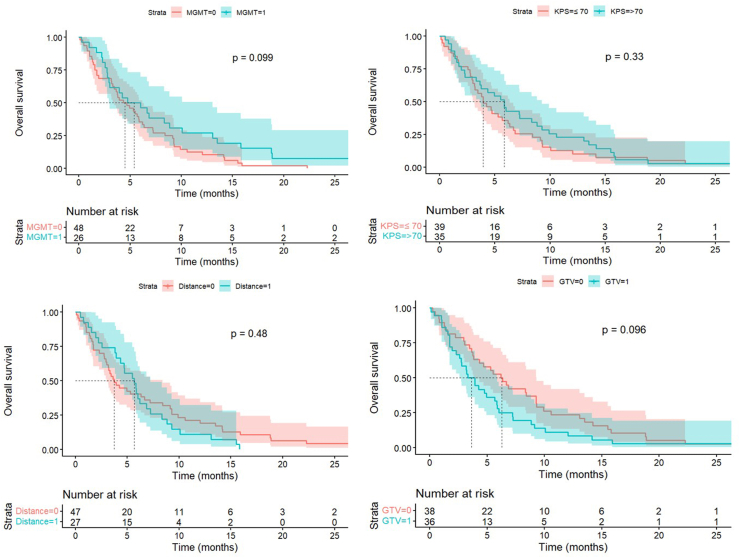
Kaplan–Meier overall survival curves stratified by selected clinical and radiological variables. Survival curves are shown according to (A) MGMT promoter methylation status, (B) Karnofsky Performance Status (KPS ≤70 vs > 70), (C) tumor proximity to the internal capsule (IC < 1 cm vs ≥ 1 cm), and (D) gross tumor volume (GTV below vs above the cohort median of 18.85 cm^3^). Median overall survival with corresponding interquartile ranges (IQR) is reported for each group. Log-rank test p-values are shown for each comparison. KPS Karnofsky Performance Status; IC Internal Capsule, GTV Gross Tumor Volume; IQR Interquartile Range.

We assessed several variables previously identified in the literature as potential risk factors in GB patients, aiming to determine their potential prognostic value in patients undergoing STX biopsy. In our cohort, median OS tended to be lower in patients with unmethylated MGMT, KPS <70, proximity to the IC < 1 cm, and larger GTV, although these differences were not statistically significant (Fig. 3). In particular, patients with unmethylated MGMT showed a median OS of 4.67 months [IQR = 1.73; 9.13], and patients with a KPS <70 had a median OS of 3.93 months [IQR = 2.57; 8.40]. Individuals with tumors located within 1 cm from the IC had a median OS of 3.73 months [IQR = 1.73; 9.90]. Patients with tumors with GTV>18.8 cm^3^ (the median of our cohort) had a median OS of 3.93 months [IQR = 1.73; 7.20]. In.

**Fig. 4:**
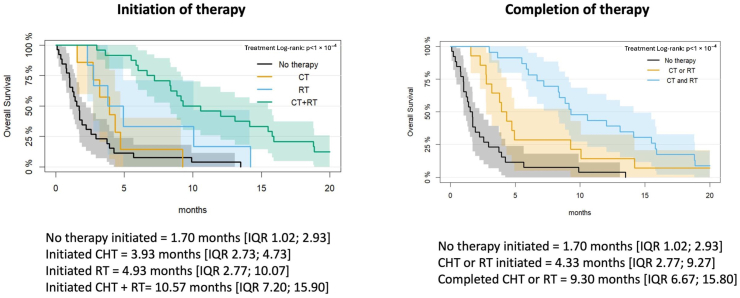
Kaplan–Meier overall survival curves according to initiation and completion of oncological treatment. (A) Overall survival stratified by initiation of treatment: no therapy, chemotherapy (CHT) alone, radiotherapy (RT) alone, and combined chemoradiotherapy (CHT + RT). (B) Overall survival stratified by treatment completion: no therapy initiated, treatment initiated but not completed (CHT or RT), and completion of at least one oncological treatment. Median overall survival with corresponding interquartile ranges (IQR) is reported for each group. Log-rank test p-values are shown for group comparisons.CHT Chemotherapy; RT Radiotherapy; OS Overall Survival; IQR Interquartile Range.

Regarding the impact of the treatment on OS, patients who did not start any form of treatment had a median OS of 1.70 months [IQR = 1.02; 2.93], those who underwent CHT only had a median OS of 3.93 months [IQR 2.73; 4.73], RT only group had a median OS of 4.93 months [IQR 2.77; 10.07], concomitant treatment according to Stupp's schedule had a median OS of 10.57 months (p < 0.001) [IQR 7.20; 15.90] (Fig. 4).

When considering treatment completion, the median OS was lower for patients who did not complete CHT or RT (4.33 months [IQR 2.77; 9.27]) than for those who completed at least one treatment (9.30 months [IQR 6.67; 15.80 p < 0.001]).

**Fig. 5:**
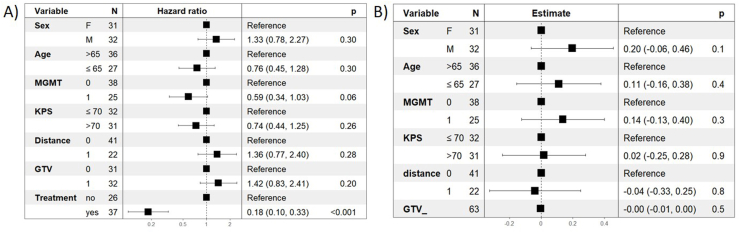
Results of Cox (panel A) and logistic (panel B) regression models to evaluate the impact of various demographic and clinical risk factors on overall survival (OS) and access to treatment, respectively. Effect estimates are reported as hazard ratios (HR) for the Cox model (A) and regression coefficients for the logistic model (B), with corresponding 95% confidence intervals and p-values. KPS Karnofsky Performance Status; GTV Gross Tumor Volume; OS Overall Survival.

Subsequently, a Cox proportional regression model was performed to evaluate the association between selected demographic, clinical and radiological variables and overall survival (Fig. 5A). The model included sex, age (above or below 65 years), MGMT promoter methylation status (methylated or unmethylated), KPS (≤70 or >70), distance from the IC (within or beyond 1 cm), GTV (above or below the median value of our cohort, i.e., 18.85 cm^3^), and starting of any form of treatment (CHT or RT). Initiation of at least one form of treatment was the only variable significantly associated with improved overall survival (p < 0.001).

Finally, a logistic regression model was used to assess factors associated with the likelihood of receiving any form of treatment (Fig. 5B). This model included sex, age, MGMT promoter methylation status, KPS, distance from the IC, and GTV. None of these variables were significantly associated with receiving adjuvant treatments.

## 4 Discussion

Many authors have extensively described neurosurgical and neuro-oncological aspects of GB; however few studies are focused on unresectable GBs (Weller et al., 2023). Most of the literature is about technical aspects of brain biopsies and their complications rather than surgical indications and outcomes of GB patients after surgery.

Brain biopsy is generally regarded as a minor, easy to perform, low-cost procedure for both the patient and the healthcare system. However, it is often performed just for further confirmation of a radiological diagnosis of GB. In a setting of short resources and increased health system costs, it may be interesting to understand.

• complication rates for confirmatory biopsies for GB;
• what factors can affect access to treatments after a brain biopsy;
• what influence can a brain biopsy have on the kind of therapy delivered.

Such information is not available in literature for this subset of patients that undergo a merely confirmatory STX. The existing literature primarily focuses on surgical complications of STX biopsy, often including heterogeneous cohorts encompassing both neoplastic and non-neoplastic lesions (Sciortino et al., 2019; Riche et al., 2021; McGirt et al., 2005; Malone et al., 2015; Tilgner et al., 2005). Even when access to care and neuro-oncological outcomes in patients with GB undergoing biopsy are addressed, analyses are frequently based on histopathological diagnosis (Halaj et al., 2024; Weller et al., 2023). As a result, patients with clear radiological diagnosis of GB who undergo STX biopsy for diagnostic confirmation, as in the present study, are often not specifically considered.

### 4.1 Brain biopsy and post-operative complications

Complications of a STX biopsy can be divided into three groups: haemorrhages (including major bleedings), neurological deficits and inconclusive histological diagnosis. Postoperative complication rate reported in literature ranges from 1% to 13% (Sciortino et al., 2019; Riche et al., 2021; McGirt et al., 2005; Malone et al., 2015; Tilgner et al., 2005).

In a recent systematic review a lower rate of major complications (<1%) was found, but a higher rate of asymptomatic radiological complications (up to 59% of patients) was noted (Riche et al., 2021). The same author reported a 0-3.9% of new post-operative neurological deficits; with a higher risk for brainstem GB (nearly 20% of patients had a neurological deterioration) (Malone et al., 2015). In a meta-analysis of 2400 patients, brain biopsy had a post-operative morbidity of 1.3% up to 27.8% and a post-operative mortality ranging from 1.2% to 3.9% (Ungar et al., 2022). New-onset deficits after frame-based biopsy ranged from 2.8% to 13.9%. Post-surgical complications are not technique-related (frame-based or frameless) (Ungar et al., 2022; Dhawan et al., 2019). Complications rates for resection ranged instead from 15% to 24% (Chaichana et al., 2011; Kommers et al., 2021; Kita et al., 2009). Complications rate reported for GB patients in the Dutch Quality Registry Neurosurgery accounted for 20% on 2271 resections and 11% on 1017 biopsies (Kommers et al., 2021). Complication rate in our patients was similar to the reported rates in literature (10.8%) and included two (2.7%) severe complications (neurological deterioration leading to mortality within 30 days), which were slightly higher than the data previously reported.

In one case, a patient received an inconclusive biopsy result and had to undergo a second biopsy. After the second procedure, he was submitted to adjuvant therapy while awaiting the report of the new biopsy in order to avoid waste of time.

### 4.2 STX biopsy indications and prognostic factors

Our objective was to identify potential risk factors, assess prognosis and examine access to treatments. Current knowledge indicates that prognostic factors for GB include age, preoperative deficits, tumour location and the feasibility of safe surgical resection (Chaichana et al., 2011; Kita et al., 2009; Bruno et al., 2022; Amsbaugh et al., 2017; Riche et al., 2022).

The 2021 guidelines from EANO (Weller et al., 2021) recommend that clinical decisions made without histological diagnosis should be restricted to exceptional circumstances, albeit with a low level of recommendation primarily based on expert opinion. Similar recommendations are found in other guidelines (Weller et al., 2021; Olson et al., 2019; Stupp et al., 2010). Histology remains the gold standard for GB diagnosis and it is considered important when counselling a patient regarding adjuvant therapies and disease prognosis (Olson et al., 2019). However, performing a biopsy for diagnosis confirmation has its drawbacks, including potential risks and costs—both in terms of decreased quality of life and healthcare resource utilization—that may not be justified. For example, it is reported in a work by Riche et al. that management of any patient suffering a symptomatic complication for a brain biopsy has an average extra cost of about 35000$^50^. Guidelines do not make exceptions even to patients who are considered unfit for adjuvant therapy and will undergo comfort care only (Stupp et al., 2010).

We therefore evaluated the impact of selected clinical, radiological and histological parameters on OS and on the likelihood of initiating any form of treatment, both individually and through a logistic regression model, to assess their potential role in guiding surgical decision-making. In our patient cohort, unmethylated MGMT status, a KPS ≤70, proximity to the IC within 1 cm, and a GTV ≥18.85 cm^3^ were all associated with worse OS, although none reached statistical significance (Di Cristofori et al., 2017; Sadeghi et al., 2008). This is likely due to the considerable clinical and radiological heterogeneity of the patients included in the study; but they may help defining the population of patients that may not benefit from a confirmatory brain biopsy.

### 4.3 Neuro-oncological outcomes after brain biopsy

Few authors have focused on the neuro-oncological outcomes of patients with GB undergoing biopsies. In many cases, particularly among patients with severe clinical impairments (e.g. cognitive deficits) or low KPS, oncological treatments are either not initiated or not completed despite histological confirmation, as reflected by the high proportion of biopsied GB patients who did not receive active neuro-oncological therapies.

In a previous report, median OS of GB patients undergoing Stupp protocol after biopsy was 6.6 months, but 70% of patients did not receive any form of adjuvant therapy (Halaj et al., 2024). Median OS after biopsy for GB confirmation was short (4.1 months) in elderly patients with low median KPS (<80); while longer term survival was predicted by good performance status, high KPS (>80), MGMT hypermethylation and small GTV (Weller et al., 2023).

In the latter study, clinical status was the main determinant of access to further therapy and not molecular features.

In our cohort, patients who did not receive any oncological treatment had a median overall survival (OS) of 1.7 months, which was significantly higher in patients who started any sort of treatment [3.9 months for RT only, 4.9 for CHT only, 10.6 in patient who underwent both (p value < 0.001)].

Regarding access to treatment, over one-third of patients did not receive any therapeutic intervention due to advanced radiological and clinical conditions or rapid clinical deterioration following STX biopsy (Pichardo-Rojas et al., 2024; Soliman et al., 2022). Furthermore 4% of patients received at least one cycle of RT and 23% of them failed to complete the treatment. In terms of CHT, 48% of patients accessed at least one cycle of TMZ while only 16% completed the first six cycles as per Stupp protocol. Several patients undergoing CHT experienced significant adverse complications like pneumonias, pulmonary embolism, sepsis, and spondylodiscitis that required hospitalization in 8 cases.

Patients undergoing biopsy only for suspected GB are, by the nature of this choice, a group of patients displaying a combination of factors (including tumor size, location, age, performance status) typically associated with poor prognosis (Pichardo-Rojas et al., 2024; Soliman et al., 2022). In the clinical practice they often demonstrate having a short therapeutic window for starting any kind of therapies before experiencing a neurological decline. A brain biopsy has not a negligible rate of complications and it does not appear to significantly inform treatment access and strategies; but it may play a role in delaying access to active treatment especially in case of complications.

In this subset of neuro-oncological patients, in whom the natural course of the disease is rapidly progressive and associated with poor prognosis, access to adjuvant therapies is crucial for both QoL and OS. However, it must be carefully balanced against the potential “loss of time” associated with the biopsy, including the waiting period between the procedure and the pathological diagnosis, as well as the possible complications related to intervention. Given the few reports about neuro-oncological outcomes after confirmatory brain biopsies for GB, our experience may be of help for clinicians in order to better inform patients with unresectable GB about post-surgical outcomes and their potential prognosis.

### 4.4 Limitations

Although all patients were treated within a neuro-oncological pathway, the retrospective nature of this study may have affected the results. On the other hand, this study was not designed and did not aim to confirm sensitivity and specificity of radiological findings to diagnose GB without histological confirmation; nor it was meant to try to change the current management of patients with unresectable GBs. Surgical indication, therapy indication and withdrawal of cares are clinical choices and thus could be consequence of unconscious bias by the treating clinicians or may be affected by the local policies. All of these factors can influence OS and might cause a self-fulfilling prophecy. Finally, our study lack of a cohort of patients treated with CHT and RT without biopsy and this represents another limitation of our study. The lack of such group of patients does not allow to draw conclusions about utility or futility of a brain biopsy for unresectable GBs. Further studies may need to introduce a control group of patients treated without a brain biopsy.

## 5 Conclusions

When dealing with brain tumors, histology still represents a firm point for multidisciplinary discussion and for counselling patients and their families. Nevertheless, biopsy for GB confirmation does not improve prognosis (e.g. opposite to resection) and might negatively impact it (e.g. due to complications). Given our findings and the existing literature, although brain biopsy is technically straightforward, the risks associated should not be dismissed, as they can negatively impact patients' neurological status. This aspect is particularly relevant for GB patients, where QoL might be more important than OS (Kommers et al., 2021; Baba and Adali, 2021; Colen and Allcut, 2012; Ståhl et al., 2022). A high complication rate (10.8%) and the high access to BCCs (42.2%) before receiving any oncological treatment should raise the question of whether brain biopsy —an invasive surgical procedure — can be avoided for decision making especially in patients who are deemed unfit to access or complete adjuvant treatment before STX biopsy (which, unlike resection, has no realistic chance of leading to clinical improvement). Our result showed that OS improved significantly only in patients who completed CHT or RT or who initiated both. Nevertheless, due to biopsy-related complications or disease progression, 37% of patients did not receive any of these treatments. In this view, further studies may be of help in order to understand how to better select patients that will benefit of a confirmatory brain biopsy for accessing adjuvant therapies. Our study is the first that looks at STX impact and outcomes not as a standalone procedure but in the context of the broader natural history of these patients. By focusing on patients with radiological diagnosis highly suggestive of GB, the present study offers specific considerations and findings. Despite the inherent limitation of reflecting the clinical experience of a single center, these results may contribute to international scientific discussion in a clinical context that is often unclear and challenging to interpret. In this setting QoL and patient-centered considerations frequently play a central role in clinical decision making. Further studies may be warranted to better inform clinical management and decision making in this often complex cases. On the other hand, our work has some limitations but it may be of help for some reflections about current practice and it can be a starting point for further studies.

In the next future, emerging diagnostic tools, such as liquid biopsy, artificial intelligence, radiomics, and advanced MRI techniques, may enable earlier diagnosis of GB, potentially reducing the need for confirmatory brain biopsies (Shukla et al., 2017; Tariciotti et al., 2022; Khristov et al., 2023; Seyhan, 2024; Ibrahim et al., 2023; Hangel et al., 2023; Hirschler et al., 2023).

## Author contributions

A.D.C., D.F., C. B. R., T.C., A.T. manuscript writing and drafting. Conceptualization<a name = "Line_manuscript_107">

F.G., S.G.: statistical analysis.

G.C., G.S., C.J.: data collection.

D.F., C.R., A.T., G.P.: figures and tables.

C.G., G.C.: manuscript editing and revision.

## Ethics

The study was performed in accordance with the ethical standards of the 1964 Helsinki declaration and its later amendments or comparable ethical standards. This study was approved by the ethic committee of Comitato Etico Territoriale Lombardia, CET, 3 under the study BIOPSIE GBM (protocol number 1045 – October 24, 2024).

All patients underwent diagnostic and therapeutic procedures approved for their specific disease and part of the current clinical practice. Each patient signed a consent form during the hospital recovery for use of clinical, histological and radiological data for research purposes according to institutional policy. All the data collected during the study were completely anonymized after collection.

## Data availability statement

Due to privacy restrictions, the raw data supporting the conclusions of this article will be made available by the authors on request if possible.

## Funding

The authors declare that no funds, grants, or other support were received during the preparation of this manuscript.

## Declaration of competing interest

The authors declare that they have no known competing financial interests or personal relationships that could have appeared to influence the work reported in this paper. Graphical abstract was created with BioRender.com (D. Ferlito 2025 - https://BioRender.com/03f0kui).

## References

[bib1] Albert N.L., Weller M., Suchorska B. (2016). Response assessment in neuro-oncology working group and european association for neuro-oncology recommendations for the clinical use of PET imaging in gliomas. Neuro Oncol..

[bib2] Almenawer S.A., Badhiwala J.H., Alhazzani W. (2015). Biopsy versus partial versus gross total resection in older patients with high-grade glioma: a systematic review and meta-analysis. Neuro Oncol..

[bib3] Amsbaugh M.J., Yusuf M.B., Gaskins J., Burton E.C., Woo S.Y. (2017). Patterns of care and predictors of adjuvant therapies in elderly patients with glioblastoma: an analysis of the national cancer data base. Cancer.

[bib4] Baba M.A., Adali N. (2021). Neurocognitive state and quality of life of patients with glioblastoma in mediterranean countries: a systematic review. Ann. Palliat. Med..

[bib5] Bauman M.M.J., Bouchal S.M., Monie D.D., Aibaidula A., Singh R., Parney I.F. (2022). Strategies, considerations, and recent advancements in the development of liquid biopsy for glioblastoma: a step towards individualized medicine in glioblastoma. Neurosurg. Focus.

[bib6] Bruno F., Pellerino A., Pronello E. (2022). Elderly gliobastoma patients: the impact of surgery and adjuvant treatments on survival: a single institution experience. Brain Sci..

[bib7] Certo F., Stummer W., Farah J.O. (2020). Supramarginal resection of glioblastoma: 5-ALA fluorescence, combined intraoperative strategies and correlation with survival. J. Neurosurg. Sci..

[bib8] Chaichana K.L., Garzon-Muvdi T., Parker S. (2011). Supratentorial glioblastoma multiforme: the role of surgical resection versus biopsy among older patients. Ann. Surg Oncol..

[bib9] Colen C.B., Allcut E. (2012). Quality of life and outcomes in glioblastoma management. Neurosurg. Clin..

[bib10] Corbin Z.A. (2019). New metabolic imaging tools in neuro-oncology. Curr. Opin. Neurol..

[bib11] Dhawan S., He Y., Bartek J.J., Alattar A.A., Chen C.C. (2019). Comparison of frame-based versus frameless intracranial stereotactic biopsy: systematic review and meta-analysis. World Neurosurg..

[bib12] Di Cristofori A., Zarino B., Fanizzi C. (2017). Analysis of factors influencing the access to concomitant chemo-radiotherapy in elderly patients with high grade gliomas: role of MMSE, age and tumor volume. J. Neuro Oncol..

[bib13] El Hachimy I., Kabelma D., Echcharef C., Hassani M., Benamar N., Hajji N. (2024). A comprehensive survey on the use of deep learning techniques in glioblastoma. Artif. Intell. Med..

[bib14] Ellingson B.M., Bendszus M., Boxerman J. (2015). Consensus recommendations for a standardized brain tumor imaging protocol in clinical trials. Neuro Oncol..

[bib15] Halaj M., Kalita O., Tuckova L., Hrabalek L., Dolezel M., Vrbkova J. (2024). Life expectancy in glioblastoma patients who had undergone stereotactic biopsy: a retrospective single-center study. Biomed. Pap. Med. Fac. Univ. Palacky Olomouc. Czechoslov..

[bib16] Hangel G., Schmitz-Abecassis B., Sollmann N. (2023). Advanced MR techniques for preoperative glioma characterization: part 2. J. Magn. Reson Imag. JMRI.

[bib17] Hegi M.E., Diserens A.C., Gorlia T. (2005). MGMT gene silencing and benefit from temozolomide in glioblastoma. N. Engl. J. Med..

[bib18] Hirschler L., Sollmann N., Schmitz-Abecassis B. (2023). Advanced MR techniques for preoperative glioma characterization: part 1. J. Magn. Reson Imag. JMRI.

[bib19] Horbinski C., Nabors L.B., Portnow J. (2023). NCCN guidelines® insights: central nervous system cancers, version 2.2022. J. Natl. Compr. Cancer Netw. JNCCN.

[bib20] Ibrahim M., Muhammad Q., Zamarud A., Eiman H., Fazal F. (2023). Navigating glioblastoma diagnosis and care: transformative pathway of artificial intelligence in integrative oncology. Cureus.

[bib21] Khristov V., Lin A., Freedman Z. (2023). Tumor-derived biomarkers in liquid biopsy of glioblastoma. World Neurosurg..

[bib22] Kickingereder P., Wiestler B., Sahm F. (2014). Primary central nervous system lymphoma and atypical glioblastoma: multiparametric differentiation by using diffusion-, perfusion-, and susceptibility-weighted MR imaging. Radiology.

[bib23] Kita D., Ciernik I.F., Vaccarella S. (2009). Age as a predictive factor in glioblastomas: population-based study. Neuroepidemiology.

[bib24] Kommers I., Ackermans L., Ardon H. (2021). Between-hospital variation in rates of complications and decline of patient performance after glioblastoma surgery in the Dutch quality registry neuro surgery. J. Neuro Oncol..

[bib25] Landriel Ibañez F.A., Hem S., Ajler P. (2011). A new classification of complications in neurosurgery. World Neurosurg..

[bib26] Laws E.R., Parney I.F., Huang W. (2003). Survival following surgery and prognostic factors for recently diagnosed malignant glioma: data from the glioma outcomes project. J. Neurosurg..

[bib27] Louis D.N., Perry A., Wesseling P. (2021). The 2021 WHO classification of tumors of the central nervous system: a summary. Neuro Oncol..

[bib28] Malone H., Yang J., Hershman D.L., Wright J.D., Bruce J.N., Neugut A.I. (2015). Complications following stereotactic needle biopsy of intracranial tumors. World Neurosurg..

[bib29] Mathon B., Le Joncour A., Bielle F. (2020). Neurological diseases of unknown etiology: brain-biopsy diagnostic yields and safety. Eur. J. Intern. Med..

[bib30] McAvoy M., Prieto P.C., Kaczmarzyk J.R. (2021). Classification of glioblastoma versus primary central nervous system lymphoma using convolutional neural networks. Sci. Rep..

[bib31] McGirt M.J., Woodworth G.F., Coon A.L. (2005). Independent predictors of morbidity after image-guided stereotactic brain biopsy: a risk assessment of 270 cases. J. Neurosurg..

[bib32] Olson J.J., Kalkanis S.N., Ryken T.C. (2019). Congress of neurological surgeons systematic review and evidence-based guidelines for the treatment of adults with metastatic brain tumors: executive summary. Neurosurgery.

[bib33] Osborn A.G., Salzman K.L., Linscott L.L. (2024).

[bib34] Pichardo-Rojas P.S., Pichardo-Rojas D., Marín-Castañeda L.A. (2024). Prognostic value of surgical resection over biopsy in elderly patients with glioblastoma: a meta-analysis. J. Neuro Oncol..

[bib35] Radbruch A., Fladt J., Kickingereder P. (2015). Pseudoprogression in patients with glioblastoma: clinical relevance despite low incidence. Neuro Oncol..

[bib36] Rajshekhar V. (2001). Current status of stereotactic biopsy. Stereotact. Funct. Neurosurg..

[bib37] Riche M., Amelot A., Peyre M., Capelle L., Carpentier A., Mathon B. (2021). Complications after frame-based stereotactic brain biopsy: a systematic review. Neurosurg. Rev..

[bib38] Riche M., Marijon P., Amelot A. (2022). Severity, timeline, and management of complications after stereotactic brain biopsy. J. Neurosurg..

[bib39] Sadeghi N., D'Haene N., Decaestecker C. (2008). Apparent diffusion coefficient and cerebral blood volume in brain gliomas: relation to tumor cell density and tumor microvessel density based on stereotactic biopsies. Am. J. Neuroradiol..

[bib40] Sanvito F., Kaufmann T.J., Cloughesy T.F., Wen P.Y., Ellingson B.M. (2023). Standardized brain tumor imaging protocols for clinical trials: current recommendations and tips for integration. Front. Radiol..

[bib41] Sciortino T., Fernandes B., Conti Nibali M. (2019). Frameless stereotactic biopsy for precision neurosurgery: diagnostic value, safety, and accuracy. Acta Neurochir..

[bib42] Seyhan A.A. (2024). Circulating liquid biopsy biomarkers in glioblastoma: advances and challenges. Int. J. Mol. Sci..

[bib43] Shukla G., Alexander G.S., Bakas S. (2017). Advanced magnetic resonance imaging in glioblastoma: a review. Chin. Clin. Oncol..

[bib44] Soliman M.A., Khan A., Azmy S. (2022). Meta-analysis of overall survival and postoperative neurologic deficits after resection or biopsy of butterfly glioblastoma. Neurosurg. Rev..

[bib45] Ståhl P., Henoch I., Smits A., Rydenhag B., Ozanne A. (2022). Quality of life in patients with glioblastoma and their relatives. Acta Neurol. Scand..

[bib46] Stupp R., Mason W.P., van den Bent M.J. (2005). Radiotherapy plus concomitant and adjuvant temozolomide for glioblastoma. N. Engl. J. Med..

[bib47] Stupp R., Hegi M.E., Mason W.P. (2009). Effects of radiotherapy with concomitant and adjuvant temozolomide versus radiotherapy alone on survival in glioblastoma in a randomised phase III study: 5-Year analysis of the EORTC-NCIC trial. Lancet Oncol..

[bib48] Stupp R., Tonn J.C., Brada M., Pentheroudakis G. (2010). High-grade malignant glioma: ESMO clinical practice guidelines for diagnosis, treatment and follow-up. Ann. Oncol. Off. J. Eur. Soc. Med. Oncol..

[bib49] Suh C.H., Kim H.S., Jung S.C., Choi C.G., Kim S.J. (2018). Perfusion MRI as a diagnostic biomarker for differentiating glioma from brain metastasis: a systematic review and meta-analysis. Eur. Radiol..

[bib50] Swinburne N.C., Schefflein J., Sakai Y. (2019). Machine learning for semi-automated classification of glioblastoma, brain metastasis and central nervous system lymphoma using magnetic resonance advanced imaging. Ann. Transl. Med..

[bib51] Syed W., Ibatullin M. (2024). Glioblastoma: overview and magnetic resonance spectroscopy analysis for treatment. Cureus.

[bib52] Tariciotti L., Caccavella V.M., Fiore G. (2022). A deep learning model for preoperative differentiation of glioblastoma, brain metastasis and primary central nervous system lymphoma: a pilot study. Front. Oncol..

[bib53] Tilgner J., Herr M., Ostertag C., Volk B. (2005). Validation of intraoperative diagnoses using smear preparations from stereotactic brain biopsies: intraoperative versus final diagnosis--influence of clinical factors. Neurosurgery.

[bib54] Ungar L., Nachum O., Zibly Z. (2022). Comparison of frame-based versus frameless image-guided intracranial stereotactic brain biopsy: a retrospective analysis of safety and efficacy. World Neurosurg..

[bib55] Wang S., Kim S., Chawla S. (2011). Differentiation between glioblastomas, solitary brain metastases, and primary cerebral lymphomas using diffusion tensor and dynamic susceptibility contrast-enhanced MR imaging. AJNR Am. J. Neuroradiol..

[bib56] Weller M., van den Bent M., Preusser M. (2021). EANO guidelines on the diagnosis and treatment of diffuse gliomas of adulthood. Nat. Rev. Clin. Oncol..

[bib57] Weller J., Katzendobler S., Niedermeyer S. (2023). Treatment benefit in patients aged 80 years or older with biopsy-proven and non-resected glioblastoma is dependent on MGMT promoter methylation status. J. Neuro Oncol..

[bib58] Wilhelm S.M., Dumas J., Adnane L. (2011). Regorafenib (BAY 73-4506): a new oral multikinase inhibitor of angiogenic, stromal and oncogenic receptor tyrosine kinases with potent preclinical antitumor activity. Int. J. Cancer.

[bib59] Wisnu Wardhana DP., Maliawan S., Mahadewa T.G.B., Rosyidi R.M., Wiranata S. (2024). Radiomic features as artificial intelligence prognostic models in glioblastoma: a systematic review and meta-analysis. Diagn Basel Switz..

[bib60] Xia W., Hu B., Li H. (2021). Deep learning for automatic differential diagnosis of primary central nervous system lymphoma and glioblastoma: multi-parametric magnetic resonance imaging based convolutional neural network model. J. Magn. Reson Imag. JMRI.

[bib61] Yan K., Yang K., Rich J.N. (2013). The evolving landscape of glioblastoma stem cells. Curr. Opin. Neurol..

[bib62] Zhang P., Liu B. (2020). Differentiation among glioblastomas, primary cerebral lymphomas, and solitary brain metastases using diffusion-weighted imaging and diffusion tensor imaging: a PRISMA-compliant meta-analysis. ACS Chem. Neurosci..

